# Premonitory Pain Preceding Swelling: A Distinctive Clinical Presentation of Synovial Sarcoma which may Prompt Early Detection

**DOI:** 10.1080/13577140310001644788

**Published:** 2003

**Authors:** M. V. Chandu De Silva, Ann Barrett, Robin Reid

**Affiliations:** 1 University Department of Pathology/Scottish Bone Tumour Registry Western Infirmary Glasgow G11 6NT UK; 2 University Department of Pathology Faculty of Medicine University of Colombo Sri Lanka; 3 School of Medicine Health Policy and Practice University of East Anglia Norwich NR4 7TJ UK

## Abstract

*Purpose:* The aim of this paper is to document the unusual presentation of long-standing pain at the tumour site before development of a swelling in patients with synovial sarcoma.

*Patients/methods and results:* The clinical presentation of 53 patients with synovial sarcoma was compared with 56 randomly selected patients with other sarcomas of the trunk and extremities. The two groups were similar with regard to age (*P* = 0.980), sex (*P* = 0.784) duration of symptoms (*P* = 0.697), size (*P* = 0.931) and site of tumour (*P* = 0.288). Sixteen (30.2%) patients with synovial sarcoma had pain before development of a swelling compared to two (3.6%) patients with other sarcomas (*P* < 0.001, odds ratio = 11.68, 95% confidence interval 2.53, 53.83). The mean duration of such pain was 37 months (median 24, range 6–120 months). The nature of the pain was variable. Eight patients had sharply localised tenderness. Calcification seen in the X-rays of four patients was initially misdiagnosed as benign lesions. A swelling was ultimately detected by MRI, CT, ultrasound or at physical examination. The mean duration from first presentation with pain till diagnosis of synovial sarcoma was 20 months. In three patients, at explorative surgery there was friable, vascular or necrotic tissue in the absence of a well-defined tumour mass.

*Discussion:* The occurrence of long-standing pain at the tumour site prior to development of a swelling is significantly more common with synovial sarcomas than with other sarcomas. Awareness of this unusual presentation and appropriate investigation may enable detection of synovial sarcoma at a prognostically favourable early stage.


*Sarcoma*, September/December 2003, *VOL. 7, NO. 3/4, 131–135*

Taylor & Francis healthsciences

ORIGINAL ARTICLE

### Premonitory pain preceding swelling: a distinctive clinical presentation of synovial sarcoma which may prompt early detection

M.V. CHANDU DE SILVA[1,2], ANN BARRETT[3] & ROBIN REID[1]

[1]*University Department of Pathology/Scottish Bone Tumour Registry, Western Infirmary, Glasgow G11 6NT, UK*
[2]*University Department of Pathology, Faculty of Medicine, University of Colombo, Sri Lanka*
[3]*School of Medicine, Health Policy and Practice, University of East Anglia, Norwich NR4 7TJ, UK*

**Abstract**

*Purpose:* The aim of this paper is to document the unusual presentation of long-standing pain at the tumour site before development of a swelling in patients with synovial sarcoma.
*Patients/methods and results:* The clinical presentation of 53 patients with synovial sarcoma was compared with 56 randomly selected patients with other sarcomas of the trunk and extremities. The two groups were similar with regard to age ($P=0.980$), sex ($P=0.784$) duration of symptoms ($P=0.697$), size ($P=0.931$) and site of tumour ($P=0.288$). Sixteen (30.2%) patients with synovial sarcoma had pain before development of a swelling compared to two (3.6%) patients with other sarcomas ($P<0.001$, odds ratio = 11.68, 95% confidence interval 2.53, 53.83). The mean duration of such pain was 37 months (median 24, range 6–120 months). The nature of the pain was variable. Eight patients had sharply localised tenderness. Calcification seen in the X-rays of four patients was initially misdiagnosed as benign lesions. A swelling was ultimately detected by MRI, CT, ultrasound or at physical examination. The mean duration from first presentation with pain till diagnosis of synovial sarcoma was 20 months. In three patients, at explorative surgery there was friable, vascular or necrotic tissue in the absence of a well-defined tumour mass.
*Discussion:* The occurrence of long-standing pain at the tumour site prior to development of a swelling is significantly more common with synovial sarcomas than with other sarcomas. Awareness of this unusual presentation and appropriate investigation may enable detection of synovial sarcoma at a prognostically favourable early stage.

**Key words:** *synovial sarcoma, pain, diagnosis, clinical features*

### Introduction

Synovial sarcoma is a clinically and morphologically distinct neoplasm of uncertain histogenesis mostly affecting the extremities of adolescents and young adults. It is the fourth commonest soft tissue sarcoma in adults,[1] with approximately 150 new cases a year in the UK and 650 in the USA.[2] It is regarded as an aggressive neoplasm with reported 5- and 10-year survival rates ranging, respectively, from 38 to 76%[3,4] to 20 to 63%.[5,4] The usual presenting symptom is of a swelling with or without pain.[1,3,6] The duration of symptoms ranges from 1 month to 20 years,[3] with a mean of 2.5 years.[7] We have observed that some patients with synovial sarcoma have long standing pain at the tumour site for variable periods of time prior to development of a swelling. This phenomenon has been briefly mentioned, mostly in older literature,[7,8] and in more recent isolated case reports.[9,10] In order to determine if this presentation was unique to synovial sarcoma, we compared the clinical features of 53 patients with synovial sarcoma with those of 56 patients with other types of sarcomas involving the trunk and extremities. In this report we describe the variable clinical presentations, pathological features and survival outcome of 16 patients with synovial sarcoma who had pain at the tumour site prior to development of a swelling. As numerous studies[3,5,6,11,12] have reported that a tumour size smaller than 5 cm is associated with a better prognosis in synovial sarcoma, awareness of this unusual clinical presentation and attempts to identify synovial sarcomas at an early stage may be of benefit to patients.

### Patients and methods

The clinical presentation of 53 patients with synovial sarcoma in the Scottish Bone Tumour Registry from

Correspondence to: Dr. Robin Reid, Consultant Pathologist, University Department of Pathology, Western Infirmary, Glasgow G11 6NT, United Kingdom. Tel.: +44-141-211-2055; Fax: +44-141-337-2494; E-mail: rprlm@clinmed.gla.ac.uk

1357-714X print/1369–1643 online © 2003 Taylor & Francis Ltd
DOI: 10.1080/13577140310001644788

1955 to 1999, were compared with those of 56 patients with other sarcomas involving the trunk and extremities using the chi-square test. A *P* value of less than 0.05 was considered to be statistically significant. The other sarcomas were randomly selected from sarcomas of the extremities and trunk affecting a similar age group. These comprised 13 epithelioid sarcomas, 10 clear cell sarcomas, nine myxoid liposarcomas, seven alveolar rhabdomyosarcomas, seven extraskeletal myxoid chondrosarcomas, four leiomyosarcomas, three low-grade fibromyxoid sarcomas and three malignant fibrous histiocytomas. The clinical features were obtained from the records of the tumour registry, which had detailed accounts of the clinical features. The diagnosis was confirmed by review of the histology slides. The mean survival time of patients with synovial sarcoma and the effect of initial pain on recurrence, metastases and tumour-related death was estimated by the Kaplan–Meier method and log rank test. All statistical analysis was done using SPSS version 10.0.

### Results

Sixteen out of 53 (30.2%) patients with synovial sarcoma had pain at the site of the tumour before development of a swelling, compared to two out of 56 (3.6%) patients with other types of sarcomas. This difference was statistically significant ($P < 0.001$, odds ratio = 11.68, 95% confidence interval 2.52, 53.83). There was no statistically significant difference in the patients with synovial sarcomas and other sarcomas with regard to age ($P = 0.980$, *t*-test, mean difference 0.09 years, 95% CI −7.4, 7.6), duration of symptoms ($P = 0.697$, *t*-test, mean difference −3.70 months, 95% CI –22.51, 15.1), size of tumour ($P = 0.931$, *t*-test, mean difference 0.93 cm, 95% CI –0.97, 2.83), sex (Pearson $\chi^2 = 0.075$, df = 1, $P = 0.784$) and site of tumour (Pearson $\chi^2 = 18.634$, df = 16, $P = 0.288$).

The clinical details of the 16 patients with synovial sarcoma who had pain at the site of tumour prior to a swelling are summarised in Table 1. There were 11 females and five males. The mean age of patients was 33.4 years (range 10–77 years). The mean duration of pain prior to swelling was 36.6 months (median 24, range 6–120 months).

Fourteen patients presented initially to a doctor because of pain. Two patients (cases 11 and 15) presented with a swelling but gave a history of pain at the site, prior to development of the swelling. The nature of the pain was variable. In some it was sharply localised, in others it radiated down a limb. It was described as sharp, shooting or cramp like. Movement and knocking or jarring the site usually aggravated pain. Two patients (cases 2 & 15) who later developed tumours in the thigh complained that sitting had been uncomfortable. Two patients (cases 3 & 12) with subsequent tumours in the feet, complained that bearing weight on the ankle first thing in the morning or after sitting for long periods was painful. One patient (case 7) who had a tumour in the abdominal wall presented twice to accident and emergency for pain in the right iliac fossa. She had complained of pain off and on at the site for 2 years. Eight patients had sharply localised tenderness. Case 14 was unusual as she had bilateral enucleation of eyes for retinoblastoma at 5 months and 3 years of age.

Two patients had received steroid injections without being investigated radiologically. X-rays were done in eight patients for investigation of pain. They were normal in four and showed calcification in the others. These were diagnosed as phleboliths in a haemangioma (this patient also had abnormal signals on MRI interpreted as a haemangioma), calcific tendonitis or loose bodies. In two patients X-ray and computed tomography (CT) done after appearance of the swelling showed calcification. In one patient (case 4) ultrasound, bone scan and repeated magnetic resonance imaging (MRI) had been normal (these were not available for review). In another patient (case 13) two surgical explorations failed to reveal any abnormalities. The swelling was ultimately picked up by MRI in two patients (mean tumour size 5.5 cm), ultrasound in two (mean size 4.2 cm), CT scan and ultrasound in two (mean size 7 cm) and surgical exploration in two (mean size 2.8 cm). In six a palpable mass was ultimately detected at physical examination (mean size 4 cm), while two patients noticed the swelling themselves (mean size 8.5 cm). The mean duration from first presentation to a doctor with the pain, until diagnosis of synovial sarcoma was 20 months (range 1 week to 144 months).

Twelve patients were treated by surgical excision and four by amputation. In case 1, at surgery there was an ill-defined area of calcification surrounding and invading a nerve. In case 3, there was no definite tumour mass at surgery but blood stained soft tissue was seen extending around the tibial artery. Similarly, in case 7, there was only soft necrotic material with no obvious tumour mass. One patient had preoperative and three had postoperative chemotherapy. One patient had preoperative and two had postoperative radiotherapy. Six patients had local recurrence, nine had metastases and 10 died of the disease. The mean survival period was 91 months (95% CI 55, 127). Live patients were followed up for a mean period of 73 months (range 48–144 months). One patient was alive with metastases after 54 months of follow-up. The presence of pain before onset of swelling showed no significant association with recurrence (log rank $P = 0.631$), metastases (log rank $P = 0.533$) and tumour related death (log rank $P = 0.472$).

Of the only two patients with other sarcomas who experienced pain at the site of the tumour prior to a

**Table 1.** *Clinical features of patients with synovial sarcoma who had pain preceding the onset of swelling*

| No | Age and sex | Site | Duration of pain (months) prior to swelling | Initial investigations for pain* | Detection of swelling | Initial diagnosis | Time from presentation to definitive diagnosis† | Outcome and follow-up (months)‡ |
|---|---|---|---|---|---|---|---|---|
| 1 | 11, M | Thigh | 24 | X-ray-calcification, MRI-abnormal signal | Clinical examination | Haemangioma | 17 months | AWND, 48 |
| 2 | 77, F | Thigh | 36 | X-ray-calcification | MRI | NK | 34 months | AWND, 84 |
| 3 | 23, F | Foot | 60 | X-ray-normal | MRI | NK | 24 months | AWND, 60 |
| 4 | 39, M | Foot | 65 | X-ray, bone scan, Ultrasound, MRI-normal | Clinical examination | NK | 65 months | Died, 20 |
| 5 | 53, F | Elbow | 7 | X-ray-calcification | Ultrasound | Calcific tendonitis | 9 months | AWND, 49 |
| 6 | 26, F | Buttock | 15 | Initially-none, had steroid injection | CT scan, ultrasound | Trochanteric bursitis | 8 months | Died, 40 |
| 7 | 10, F | Abdominal wall | 24 | None | CT scan, ultrasound | NK | 1 week | Died, 41 |
| 8 | 65, M | Knee | 6 | X-ray-calcification | Surgical exploration | Loose bodies | 6 months | Died, 28 |
| 9 | 45, F | Knee | 120 | X-ray-normal, had steroid injection | Surgical exploration | Chronic tendonitis | 36 months | AWND, 144 |
| 10 | 28, M | Knee | 6 | None | Ultrasound | Ruptured popliteal cyst | 2 months | Died, 12 |
| 11 | 56, F | Upper arm | 60 | None | Noticed by patient | NK | 2 weeks | Died, 37 |
| 12 | 14, F | Foot | 18 | Had steroid injection | Clinical examination | NK | 18 months | Died, 36 |
| 13 | 15, M | Knee | 84 | X-ray-normal, surgical exploration (done twice)-normal | Clinical examination | NK | 72 months | Died, 170 |
| 14 | 12, F | Thigh | Several years | None | Clinical examination | NK | Not known | Died, 44 |
| 15 | 28, F | Thigh | 18 | None | Noticed by patient | NK | 1 week | Died, 15 |
| 16 | 33, F | Knee | 48 | None | Clinical examination | Meniscal injury | 14 months | AM, 54 |

*Indicates investigations done until detection of the swelling.
†Indicates time from first presentation to a doctor until histological diagnosis.
‡Indicates period of follow-up from definitive diagnosis.
AWND, alive and well with no disease; AM, alive with metastases; NK, not known.
All the patients who died did so due to the synovial sarcoma.

swelling, one was a 10-year-old boy who had an epithelioid sarcoma of the hand and the other was a 78-year-old man who had an extraskeletal myxoid chondrosarcoma of the axilla.

### Discussion

This study shows that patients with synovial sarcomas are 12 times more likely ($P<0.001$, odds ratio = 11.68, 95% confidence interval 2.52, 53.83) to have pain at the site of the tumour prior to development of a swelling, than patients with other sarcomas affecting the extremities and trunk. The occurrence of sharply localised pain usually associated with tenderness, necessitating a visit to a doctor in 30.2% of the patients with synovial sarcoma in our tumour registry, made us give credence to this unusual presentation.

Slightly more than half the patients with synovial sarcoma who present with a swelling may have associated pain and tenderness.[1,7] In the series reported by Wright *et al.*, 137 out of 180 patients had pain arising directly from the lesion while 27 had referred pain due to pressure.[3] Such pain, which is also seen with other sarcomas, is probably due to expansion, stretching and destruction of surrounding tissues and haemorrhage and necrosis within the tumour. The presence of pain preceding the swelling in synovial sarcoma has not been described in most previous large studies. In the series of 134 synovial sarcomas reported by Cadman *et al.*,[7] 26.9% had pain or point tenderness for periods ranging from 1 month to 18 years (mean 3.5 years) prior to appearance of a palpable mass. Haagensen and Stout,[8] mentioned this unusual presentation in their series and commented that the knee was explored in a number of patients, although details are not provided. Ours is the first large series to describe the clinical presentation, histological features and outcome of patients with synovial sarcoma who had pain prior to development of a swelling.

Awareness of this presentation and prompt investigation may enable detection of synovial sarcoma at an early stage. This may be of benefit to patients as numerous studies have reported a better prognosis with synovial sarcomas measuring less than 5 cm in diameter.[3,5,6,11,12] In our series, the patients who presented with pain preceding the swelling did not have a better survival than those without preceding pain. This is probably because diagnosis was delayed in most patients with a mean time of 20 months from first presentation to a doctor. Calcification in X-rays though detected early was misdiagnosed as calcific tendonitis or loose bodies. Synovial sarcoma should always be considered in the differential diagnosis of a soft tissue mass with amorphous calcification.[13] Calcification in synovial sarcoma may manifest as fine stippling, small spotty radiopacities or as more dense areas.[1,14] In our series, other than in one patient, ultrasound, CT scan or MRI whenever they were performed helped to detect a swelling. CT and MRI do not provide a specific or diagnostic picture but will show a para-articular heterogeneous septate mass.[1,15] Previous studies[8] have mentioned surgical exploration failing to detect swellings, as was seen in one of our patients. It is noteworthy that in our series, in three patients there was no obvious tumour mass at surgical exploration. The tissues biopsied were described as ‘necrotic material’ or ‘haemorrhagic soft tissue’. Synovial sarcomas can manifest as poorly circumscribed variegated areas with a friable or shaggy appearance with haemorrhage, necrosis or cyst formation.[1] Thus it is important to biopsy such abnormal looking tissue, especially in the context of long-standing unexplained pain. Ichinose *et al.* have described a patient who presented with a severe spot of tenderness on the thigh.[9] A biopsy of the area showed a microscopic synovial sarcoma in the fascia just deep to the subcutis.

The mechanism for this premonitory pain is difficult to explain. As it occurs at a time when the tumours are small, pressure effects on the surrounding tissues or haemorrhage or necrosis within the tumour are unlikely to be major causes of pain as in the case of other sarcomas presenting as swellings associated with pain. As the synovial sarcomas and the other sarcomas in our study were similar in respect to age and sex of patient, duration of symptoms and site and size of tumour, the pain seems to be intrinsically related to the biological behaviour of synovial sarcoma. It may be due to release of cytokines, prostaglandins and other inflammatory mediators by the tumour and requires further investigation. Synovial sarcoma cells stimulated by interleukin-1$\beta$ have been shown to produce various cytokines including tumour necrosis factor $\alpha$ *in vitro*[16] and there is a report of an interleukin-1$\alpha$ producing synovial sarcoma in a patient who had prolonged fever.[17] Conversely, one study has shown that prostaglandin ($PGE_2$) production by synovial sarcoma was low.[18]

The possibility of detecting soft tissue sarcomas at a pretumour stage is a challenging prospect. The use of CT-guided core needle biopsy may be particularly useful if an abnormal area is detected by CT or MRI. As pain related to joints is a common symptom, it will not be feasible to investigate all such patients by CT or MRI. Prudent, appropriately timed, use of radiological investigations in patients with persistent unexplained sharply localised pain may help to detect synovial sarcomas at a stage when they are associated with a better prognosis.

### References

1. Weiss SW, Goldblum JR. *Enzinger and Weiss’s Soft Tissue Tumours*. 4th edition. St Louis, MO: Mosby, 2001: 1483–509.

2. Fisher C. Synovial sarcoma. *Curr Diagn Pathol.* 1994; 1: 13–8.
3. Wright PH, Sim FH, Soule EH, Taylor WF. Synovial sarcoma. *J Bone Joint Surg* (*Am*) 1982; 64A: 112–22.
4. Mullen JR, Zagars GK. Synovial sarcoma-outcome following conservation surgery and radiotherapy. *Radiother Oncol* 1994; 33: 23–30.
5. Oda Y, Hashimoto H, Tsuneyoshi M, Takeshita S. Survival in synovial sarcoma. A multivariate study of prognostic factors with special emphasis on the comparison between early death and long term survival. *Am J Surg Pathol* 1993; 17: 35–44.
6. Brodsky JT, Burt ME, Hadju SI, Casper ES, Brennan MF. Tendosynovial sarcoma. Clinicopathological features, treatment and prognosis. *Cancer* 1992; 70: 484–9.
7. Cadman NL, Soule EH, Kelly PJ. Synovial sarcoma: an analysis of 134 tumours. *Cancer* 1965; 18: 613–27.
8. Haagenson CD. Stout AP. Synovial sarcoma. *Ann Surg* 1944; 120: 826–42.
9. Ichinose H, Derbes VJ, Hoerner H. Cutaneous pain without tumor: a manifestation of occult synovioma. *Cutis* 1978; 21: 74–8.
10. Ichinose H, Wickstrom JK, Hoerner HE, Derbes VL. The early clinical presentation of synovial sarcoma. *Clin Orthop* 1979; 142: 185–9.
11. Bergh P, Meis-Kindblom JM, Gherlinzoni F, Berlin O, Bacchini P, Bertoni F, Gunterberg B, Kindblom LS. Synovial sarcoma: identification of low and high-risk groups. *Cancer* 1999; 85: 2596–607.
12. el-Naggar AK, Ayala AG, Abdul Karim FW, McLemore D, Ballance WW, Garnsey L, Ro JY, Batsakis JG. Synovial sarcoma. A DNA flow cytometric study. *Cancer* 1990; 65: 2295–300.
13. Moser RP, Parrish WM. Radiologic evaluation of soft tissue tumours. In: Weiss SW, Goldblum JR, eds. *Enzinger and Weiss's Soft Tissue Tumours*. 4th edition. St Louis, MO: Mosby, 2001: 67.
14. Maxwell JR, Yao L, Eckardt JJ, Doberneck SA. Case report 878: densely calcifying synovial sarcoma of the hip metastatic to the lungs. *Skeletal Radiol* 1994; 23: 673–5.
15. Hirsch RJ, Yousem DM, Loevner LA, Montone KT, Chalian AA, Hayden RE, Weinstein GS. Synovial sarcomas of the head and neck: MR findings. *AJR Am J Roentgenol* 1997; 169: 1185–8.
16. Tsuji F, Oki K, Senda T, Horiuchi M, Mita S. Effects of mitogen-activated protein kinase inhibitors or phosphodiesterase inhibitors on interleukin-1-induced cytokines production in synovium-derived cells. *Immunol Lett* 1999; 68: 275–9.
17. Osaka S, Fujimoto Y, Yamazaki H, Suzuki K, Sawada S, Hayashi N. Interleukin-1 alpha producing synovial sarcoma with prolonged fever: a case report. *Jpn J Clin Oncol* 1998; 28: 436–40.
18. Knazek RA, Yee CL, Costa J. Prostaglandin and hydroxyeicosatetraenoic acid synthesis by human mesenchymal tumors. *Int J Cancer* 1985; 36: 143–52.

## References

[PDF_00403] Bergh P., Meis-Kindblom J. M., Gherlinzoni F., Berlin O., Bacchini P., Bertoni F., Gunterberg B., Kindblom L. G. (1999). Synovial sarcoma: identification of low and high risk groups.. Cancer.

[PDF_00390] Brodsky J. T., Burt M. E., Hajdu S. I., Casper E. S., Brennan M. F. (1992). Tendosynovial sarcoma. Clinicopathologic features, treatment, and prognosis.. Cancer.

[PDF_00393] CADMAN N. L., SOULE E. H., KELLY P. J. (1965). SYNOVIAL SARCOMA; AN ANALYSIS OF 34 TUMORS.. Cancer.

[PDF_00395] Haagensen C. D., Stout A. P. (1944). Synovial Sarcoma.. Ann Surg.

[PDF_00419] Hirsch R. J., Yousem D. M., Loevner L. A., Montone K. T., Chalian A. A., Hayden R. E., Weinstein G. S. (1997). Synovial sarcomas of the head and neck: MR findings.. AJR Am J Roentgenol.

[PDF_00397] Ichinose H., Derbes V. J., Hoerner H. (1978). Cutaneous pain without tumor: a manifestation of occult synovioma.. Cutis.

[PDF_00400] Ichinose H., Wickstrom J. K., Hoerner H. E., Derbes V. L. (1979). The early clinical presentation of synovial sarcoma.. Clin Orthop Relat Res.

[PDF_00432] Knazek R. A., Yee C. L., Costa J. (1985). Prostaglandin and hydroxyeicosatetraenoic acid synthesis by human mesenchymal tumors.. Int J Cancer.

[PDF_00415] Maxwell J. R., Yao L., Eckardt J. J., Doberneck S. A. (1994). Case report 878: Densely calcifying synovial sarcoma of the hip metastatic to the lungs.. Skeletal Radiol.

[PDF_00382] Mullen J. R., Zagars G. K. (1994). Synovial sarcoma outcome following conservation surgery and radiotherapy.. Radiother Oncol.

[PDF_00385] Oda Y., Hashimoto H., Tsuneyoshi M., Takeshita S. (1993). Survival in synovial sarcoma. A multivariate study of prognostic factors with special emphasis on the comparison between early death and long-term survival.. Am J Surg Pathol.

[PDF_00428] Osaka S., Fujimoto Y., Yamazaki H., Suzuki K., Sawada S., Hayashi N. (1998). Interleukin-1 alpha producing synovial sarcoma with prolonged fever: a case report.. Jpn J Clin Oncol.

[PDF_00423] Tsuji F., Oki K., Senda T., Horiuchi M., Mita S. (1999). Effects of mitogen-activated protein kinase inhibitors or phosphodiesterase inhibitors on interleukin-1-induced cytokines production in synovium-derived cells.. Immunol Lett.

[PDF_00380] Wright P. H., Sim F. H., Soule E. H., Taylor W. F. (1982). Synovial sarcoma.. J Bone Joint Surg Am.

[PDF_00407] el-Naggar A. K., Ayala A. G., Abdul-Karim F. W., McLemore D., Ballance W. W., Garnsey L., Ro J. Y., Batsakis J. G. (1990). Synovial sarcoma. A DNA flow cytometric study.. Cancer.

